# Neutrophil and Eosinophil DNA Extracellular Trap Formation: Lessons From Pathogenic Fungi

**DOI:** 10.3389/fmicb.2021.634043

**Published:** 2021-02-18

**Authors:** Juliana da Costa Silva, Glaucia de Azevedo Thompson-Souza, Marina Valente Barroso, Josiane Sabbadini Neves, Rodrigo Tinoco Figueiredo

**Affiliations:** ^1^Institute of Microbiology Paulo de Góes, Federal University of Rio de Janeiro, Rio de Janeiro, Brazil; ^2^Institute of Biomedical Sciences, Federal University of Rio de Janeiro, Rio de Janeiro, Brazil; ^3^Campus Duque de Caxias, Federal University of Rio de Janeiro, Rio de Janeiro, Brazil

**Keywords:** neutrophil extracellular traps, eosinophil extracellular traps, pathogenic fungi, neutrophils, eosinophils

## Abstract

Fungal infections represent a worldwide health problem. Fungal pathogens are responsible for a variety of conditions, including superficial diseases, allergic pathologies and potentially lethal invasive infections. Neutrophils and eosinophils have been implicated as effector cells in several pathologies. Neutrophils are major effector cells involved in the control of fungal infections and exhibit a plethora of antifungal mechanisms, such as phagocytosis, reactive oxygen species production, degranulation, extracellular vesicle formation, and DNA extracellular trap (ET) release. Eosinophils are polymorphonuclear cells classically implicated as effector cells in the pathogenesis of allergic diseases and helminthic infections, although their roles as immunomodulatory players in both innate and adaptive immunity are currently recognized. Eosinophils are also endowed with antifungal activities and are abundantly found in allergic conditions associated with fungal colonization and sensitization. Neutrophils and eosinophils have been demonstrated to release their nuclear and mitochondrial DNA in response to many pathogens and pro-inflammatory stimuli. ETs have been implicated in the killing and control of many pathogens, as well as in promoting inflammation and tissue damage. The formation of ETs by neutrophils and eosinophils has been described in response to pathogenic fungi. Here, we provide an overview of the mechanisms involved in the release of neutrophil and eosinophil ETs in response to fungal pathogens. General implications for understanding the formation of ETs and the roles of ETs in fungal infections are discussed.

## Introduction

Fungi represent major human pathogens. Estimates indicate that approximately 300 different fungal species are pathogenic to humans (Taylor et al., 2001; Wardeh et al., 2015), with fungal infections contributing to nearly 1.5–1.6 million deaths annually (Brown et al., 2012). The number of individuals affected by fungal diseases is increasing worldwide. Risk factors for the development of severe invasive mycoses include immunosuppression, cancer, bone marrow or solid organ transplantation, and the aging (Suleyman and Alangaden, 2016). Although fungal infections contribute greatly to human morbidity and mortality, the true magnitude of these diseases in humans is unknown due to underreporting or misdiagnosis (Vallabhaneni et al., 2016). In cases of invasive fungal infections, the mortality rate of patients can exceed 50% (Brown et al., 2012; Vallabhaneni et al., 2016). In addition to acting as causative agents of infections, fungi are also implicated in allergic pathologies. Exposure to environmental fungi and their antigens promotes allergic pathologies such as asthma and rhinitis (Knutsen et al., 2012), and fungal colonization is a common complication of asthma resulting in allergic bronchopulmonary mycoses (ABPMs) (Agarwal and Chakrabarti, 2013; Ishiguro et al., 2014).

Neutrophils and eosinophils have been implicated as effector cells in infections caused by fungal pathogens and in diseases associated with allergic sensitization to fungi (Yoon et al., 2008; Lilly et al., 2014; Gazendam et al., 2016a; Figueiredo and Neves, 2018). Conditions resulting in neutropenia or deficiencies in neutrophil responses are major risk factors for the development of severe systemic mycoses (Lilic, 2012; Gazendam et al., 2016a). Neutrophils and eosinophils are promptly recruited to inflammatory settings where their activation contributes to tissue damage and immunopathology through the release of toxic components of their granules, the generation of reactive oxygen species (ROS), the production of inflammatory mediators and the formation of DNA extracellular traps (ETs) (Amulic et al., 2012; Fulkerson and Rothenberg, 2013).

Neutrophils are peripheral blood cells of the myeloid lineage and the most abundant leukocytes in human blood, constituting approximately 70% of the leukocytes in human peripheral blood (and approximately 30% of the leukocytes in mouse blood). Neutrophils can be distinguished from other granulocytes by the absence of granule staining upon exposure to acidic or basic dyes (neutral property for which these cells were named), and in addition, the presence of multilobed nuclei allows their identification as polymorphonuclear leukocytes (PMNs) (Amulic et al., 2012). Neutrophils are essential for the clearance of fungal pathogens. Neutrophils kill fungi by a variety of mechanisms, including degranulation, phagocytosis, oxidative burst, the release of extracellular vesicles, and the formation of neutrophil DNA extracellular traps (NETs) (Urban et al., 2006; Gazendam et al., 2016a; Shopova et al., 2020). Neutrophils express pattern recognition receptors (PRRs) involved in the recognition of fungi, such as Toll-like receptor (TLR) 2 and TLR4, C-type lectin receptors (CLRs), such as Dectin-1, Dectin-2, and Mincle, the β_2_-integrin Mac-1 (macrophage-1 antigen, also known as complement receptor 3/CR3, α_M_β_2_ integrin or CD11b/CD18) (Futosi et al., 2013; Patin et al., 2019). In summary, these receptors trigger signaling pathways that coordinate neutrophil responses involved in the killing of fungal pathogens (Gazendam et al., 2016a; Lehman and Segal, 2020).

Eosinophils constitute a minor leukocyte population in the bloodstream, comprising from 1 to 5% of circulating cells, and a sudden increase in eosinophil blood counts in certain pathological conditions indicates that eosinophils are linked to the onset and maintenance of inflammatory processes (Hogan et al., 2008; Fulkerson and Rothenberg, 2013). Eosinophils are characterized by their numerous cytoplasmic granules, including crystalloid granules, primary granules, and secretory vesicles. Among these, crystalloid granules are the largest, and unique to eosinophils, they contain a variety of preformed granule-stored proteins, including cationic proteins, such as major basic protein (MBP), which is the most abundant granular protein in eosinophils; eosinophilic peroxidase (EPO); eosinophil cationic protein (ECP); and eosinophil-derived neurotoxin (EDN) (Muniz et al., 2012; Acharya and Ackerman, 2014). The abundance of cationic proteins makes eosinophils stainable by acidic dyes, such as eosin, which can be used to distinguish them from other polymorphonuclear leukocytes. Classically, eosinophils are considered effector cells in allergic pathologies and helminthic infections and have been implicated in tissue damage in allergic inflammatory pathologies. However, evolving knowledge in the field has revealed that eosinophils exhibit inflammatory and immunomodulatory functions and play roles in tissue remodeling (Lee et al., 2004; Jacobsen et al., 2008; Fulkerson and Rothenberg, 2013).

The increase in eosinophil blood counts, as well as tissue eosinophilia in allergic diseases associated with fungal sensitization/colonization, has been widely recognized (Knutsen et al., 2012; Figueiredo and Neves, 2018). Exposure and sensitization to fungal allergens is an important factor in patients with respiratory allergies, and in this context, fungi play important roles in the development, severity and persistence of allergic lung diseases, especially asthma (Knutsen et al., 2012). ABPMs are characterized by robust inflammation due to fungal colonization of the airways, particularly in patients with asthma or cystic fibrosis (Knutsen et al., 2012). One of the main characteristics indicating a diagnosis of ABPM is eosinophilia, in addition to increased serum IgE levels and colonization of the airways by fungi (Asano et al., 2020). Although ABPMs have a profile of a Th2 inflammatory response with eosinophilic infiltrates, the nature of the interactions between eosinophils and fungi is unclear, and the participation of eosinophils in fungal infections continues to be extensively discussed, as eosinophils played no role in certain aspects of the pulmonary pathology in the experimental ABPM induced by *Aspergillus fumigatus* exposure (Dietschmann et al., 2020). However, whether these findings are relevant clinically or are limited to the experimental model utilized remains to be elucidated. In the last decade, new data have shown an important antifungal role of eosinophils against several species of fungi, such as *Alternaria alternata, Cryptococcus neoformans*, and *A. fumigatus*, the latter being the main cause of ABPMs (Yoon et al., 2008; Garro et al., 2011; Lilly et al., 2014).

Extracellular traps are produced by neutrophils and eosinophils in response to fungal pathogens and are involved in the killing and/or entrapment of fungi (Muniz et al., 2018; Urban and Nett, 2019). Considerable effort has been dedicated to the elucidation of the mechanisms of neutrophil and eosinophil ET formation. While this work has focused on the formation of DNA extracellular traps in response to fungi, more detailed information on the basic aspects of NET and eosinophil extracellular trap (EET) formation can be found in recent publications (Mukherjee et al., 2018; Papayannopoulos, 2018). Here, we review the mechanisms by which ETs are released from neutrophils and eosinophils in response to fungal pathogens and the role of these ETs in pathologies caused by fungal infections or hypersensitivity responses to fungi and their antigens.

## Neutrophil Extracellular Traps

Takei et al. (1996) characterized a previously undiscovered process of cell death in human neutrophils stimulated with phorbol-12-myristate-13-acetate (PMA). Neutrophil death induced by PMA is characterized by morphological changes in the shape of the nucleus, chromatin decondensation, and further leakage of the nuclear envelope without markers of apoptosis or necrosis (Takei et al., 1996). Later, it was demonstrated that the PMA-induced neutrophil death represents a novel effector mechanism that culminates with the release of DNA fibers involved in the killing gram-negative and gram-positive bacteria (Brinkmann et al., 2004). These DNA web-like structures released by neutrophils are called NETs and contain histones and granular proteins such as neutrophil elastase (NE) and myeloperoxidase (MPO) (Brinkmann et al., 2004). The process involved in the NET release represents a novel type of cell death, called NETosis, characterized by the loss of the lobulated nuclear structure, fragmentation of the nuclear envelope, interaction of granular proteins with decondensed chromatin and the subsequent extrusion of nuclear content into the extracellular compartment (Fuchs et al., 2007).

However, further investigations demonstrated that NET release can also occur without membrane rupture and can occur independent of neutrophil death (Yousefi et al., 2009; Yipp et al., 2012). The origin of the DNA released has also been investigated. Neutrophils primed with granulocyte/macrophage colony-stimulating factor (GM-CSF) and then further stimulated with lipopolysaccharide (LPS) or complement factor 5a (C5a) release mitochondria-derived DNA by a process that maintains the integrity of the nuclear membrane and neutrophil viability. Mitochondrial NETs do not present nuclear components, such as lamin B or nuclear DNA; however, these structures contain mitochondrial DNA in association with granular proteins, such as NE and MPO. The release of mitochondrial NETs does not involve cell death, membrane rupture or nuclear disruption and is faster than the NET formation involving the nuclear DNA release, with mitochondrial NETs released within 15 min of stimulation in a mechanism that requires the generation of ROS by NADPH oxidase (Yousefi et al., 2009). The general contribution of mitochondrial NETs, however, is still unclear since the NETs formed in response to several relevant stimuli and pathogens are composed basically of nuclear DNA (Pilsczek et al., 2010; Kenny et al., 2017). Furthermore, in many experimental models and clinical samples, histones are associated with NETs, indicating that nuclear chromatin is the source of these NETs (Brinkmann et al., 2004; Guimarães-Costa et al., 2009; Röhm et al., 2014).

Neutrophils are terminally differentiated cells unable to initiate mitosis; however, signaling pathways involved in the mitotic program are involved in NETosis. Induction of NETosis leads to the expression of the mitotic marker Ki-67 and the phosphorylation of the retinoblastoma protein pRb, lamin A/C and serine 10 in histone 3, modifications characteristic of cell cycle entry. NET inducers, however, do not promote neutrophil DNA replication or cell division. Neutrophils express CDK4 and 6, and CDK6 is required for NET formation (Amulic et al., 2017). Thus, pathways in NETosis induction and mitosis are shared, with the activation of pre-mitotic machinery inducing a neutrophil-specific cell death program that leads to nuclear disruption and chromatin release into the extracellular environment.

Autophagy has been described as a process required for NETosis based on the observation of autophagic vesicles and the inhibition of NET formation by class III phosphatidylinositol 3-kinase (PI3K) inhibitors, such as wortmannin and 3-methyladenine (3-MA) (Mitroulis et al., 2011; Remijsen et al., 2011). The role of autophagy in the NET release, however, has not been confirmed in ATG5 mice conditionally deficient in neutrophils and eosinophils or by the pharmacological inhibition of autophagosome acidification (Germic et al., 2017). Thus, it seems that the effects of inhibitors of class III PI3Ks, such as wortmannin, reflect other pharmacological targets, such as class I PI3Ks, the activity of which is required for NETosis induced by *Leishmania amazonensis* and *A. fumigatus* (DeSouza-Vieira et al., 2016; Silva et al., 2020). Alternatively, since Germic et al. investigated the formation of ETs by a rapid mechanism demonstrated for stimuli that induce the formation of mitochondrial ETs (Germic et al., 2017), they could not exclude the participation of autophagic mechanisms in the ETosis in response to inducers that promote nuclear fragmentation and release of chromatin, by a process that takes hours to occur, resulting in cell death (Fuchs et al., 2007). Thus, it remains to be established whether autophagy is a consequence of neutrophil activation and occurs in parallel but is not involved in the mechanisms triggering ETosis, or whether autophagy is necessary under some conditions leading to ETosis, particularly ET formation originating from nuclear chromatin (Fuchs et al., 2007; Thiam et al., 2020).

Several inflammatory stimuli have been described as inducers of NET formation, including calcium ionophores, PMA, a protein kinase C activator, bacteria, fungi, protozoans, viruses, and immune complexes (Brinkmann et al., 2004; Urban et al., 2006; Bruns et al., 2010; McCormick et al., 2010; Saitoh et al., 2012; Neeli and Radic, 2013; Muraro et al., 2018; Veras et al., 2020). NETosis induced by PMA, a protein kinase C (PKC) activator, bacteria, fungi, or immune complexes, requires the activity of NADPH oxidase in a mechanism that relies on the generation of ROS (Fuchs et al., 2007; Ermert et al., 2009; Behnen et al., 2014; Kenny et al., 2017). A role for oxidants generated by the NADPH oxidase complex has been demonstrated by the impairment in NET formation by neutrophils obtained from individuals suffering from chronic granulomatous disease (CGD), which show impaired NADPH oxidase activity (Fuchs et al., 2007). The role of ROS generation downstream of NADPH oxidase activity has been supported by considerable evidence: (1) exogenous H_2_O_2_ or the extracellular generation of H_2_O_2_ by the incubation of neutrophils with glucose oxidase induces NETosis in the absence of NADPH oxidase activity, and (2) antioxidants are effective inhibitors of NET formation in response to many neutrophil activators (Fuchs et al., 2007; Kenny et al., 2017). In addition, NADPH oxidase-independent mechanisms for NET formation have been described in response to a toxin-producing *Staphylococcus aureus* strain (Pilsczek et al., 2010). NET formation by ROS-independent mechanisms has also been observed for a calcium ionophore and nigericin (Kenny et al., 2017).

In addition to the roles of ROS generated by NADPH oxidase, NETosis requires the activity of NE and MPO. Genetic deficiency of MPO results in impairment of NET release in response to PMA and other NET inducers (Metzler et al., 2011). During NETosis, NE and MPO are released from azurophilic granules into the cytosol (Papayannopoulos et al., 2010; Metzler et al., 2014). NE is then translocated to the nucleus, where it degrades histones. This process is amplified by MPO, which also shows nuclear localization upon the induction of NETosis (Papayannopoulos et al., 2010). The inhibition of NE results in impaired NET formation, and NE-deficient mice do not exhibit NETs in the lungs in an experimental model of infection by *Klebsiella pneumoniae* (Papayannopoulos et al., 2010). The cellular compartmentalization of NE during neutrophil activation is a determinant of the induction of NETosis. Recognition of pathogens under conditions favorable to phagocytosis does not result in NETosis, while fungal morphotypes, such as hyphae, that are not phagocytosed by neutrophils induce NET formation (Branzk et al., 2014). The trigger for NET release has been attributed to the release of NE from azurophilic granules to the cytosol in response to the recognition of pathogens in the absence of internalization by neutrophils. In contrast, phagocytosis results in the targeting of elastase to phagosomes, which turns off the NETosis program due to the reduction in the amount of cytosolic NE required for nuclear translocation and histone degradation (Branzk et al., 2014).

Histone citrullination by peptidyl arginine deiminase 4 (PAD4) has also been implicated in the induction of NETosis (Wang et al., 2009; Li et al., 2010). Peptidyl arginine deiminases constitute a group of enzymes that modify arginine residues in polypeptide chains by deimination or demethylimination, resulting in the formation of citrulline (Wang and Wang, 2013). PAD4 is highly expressed in neutrophils, and stimuli, such as calcium ionophores, TNF and LPS, promote histone citrullination (Neeli et al., 2008; Wang et al., 2009). The role of PAD4 in NET formation was first revealed by the observation that HL-60 leukemia cells when differentiated to a neutrophil-like phenotype undergo histone citrullination in response to a calcium ionophore that is associated with ET formation (Wang et al., 2009). The release of ETs by the HL-60 cell line requires the activity of PAD4 since its inhibition results in the absence of ET formation and histone citrullination. The role of PAD4 in the HL-60 model was attributed to chromatin decompaction by histone citrullination and was also observed when HL-60 cells were primed with CXCL8 followed by stimulation with *Shigella flexneri* (Wang et al., 2009). Following these observations, the involvement of PAD4 in the process of NET formation has been described in many experimental settings (Li et al., 2010; Lewis et al., 2015). A recent study demonstrated that NETosis proceeds through actin cytoskeleton dismantling, nuclear envelope rupture and chromatin decondensation that precede DNA release, and nuclear rupture requires PAD4 nuclear localization and activity (Thiam et al., 2020). The relevance of PAD4-mediated histone citrullination for NET formation has been demonstrated in PAD4-KO mice (Li et al., 2010). PAD4-deficient neutrophils do not show histone citrullination or NET release when stimulated with calcium ionophores, LPS, PMA, H_2_O_2_, or *S. flexneri*, and PAD4-KO mice show an increased susceptibility to a DNAse-deficient *Streptococcus pyogenes* strain (Li et al., 2010). A role for PAD4 in the formation of NETs in experimental models of inflammatory pathologies and infections has also been demonstrated (Hemmers et al., 2011; Martinod et al., 2013, 2017).

However, the role of PAD4-mediated histone citrullination as a universal promoter of NETosis has been questioned. PMA does not promote histone citrullination in human neutrophils under conditions in which NETs are formed (Neeli and Radic, 2013). Furthermore, PMA inhibits the histone citrullination induced by calcium ionophores without interfering with the NET release promoted by these stimuli (Neeli and Radic, 2013). NETosis in response to many pathogens, such as group B streptococci, *K. pneumoniae* and fungi, does not require PAD4-mediated histone citrullination (Kenny et al., 2017; Claushuis et al., 2018; Guiducci et al., 2018; Silva et al., 2020; Thompson-Souza et al., 2020). PAD4-dependent histone citrullination has been suggested to be an essential process for the induction of NETosis in response to calcium ionophores (Li et al., 2010; Lewis et al., 2015), but this supposition has been questioned (Kenny et al., 2017). The reasons for the discrepancies in the roles of PAD4 in NETosis are not clear. Although the data seem to indicate that PAD4-mediated histone citrullination is the mechanism responsible for the induction of NETosis, PAD4 can mediate other signaling mechanisms, possibly through the citrullination of non-histone proteins (Loos et al., 2008, 2009; Proost et al., 2008; Jang et al., 2015; Sun et al., 2017). Thus, other roles for PAD4 that do not involve chromatin decompaction by histone citrullination may be involved indirectly in the induction of NETosis *in vitro* and *in vivo*. Alternatively, the involvement of PAD4-mediated histone citrullination in the NETosis must not reflect a common process for NET formation but must be dispensable depending on the NET inducer.

While NADPH oxidase-induced ROS generation and PAD4 histone citrullination have been identified as downstream mediators of NET formation, the receptors and upstream signaling pathways critical for triggering NETosis are less understood. PMA has been used as a prototypical NET inducer requiring ROS generation by the NADPH oxidase complex (Fuchs et al., 2007). PMA-induced NETosis involves the activity of conventional PKCβ isoforms (Neeli and Radic, 2013). In contrast, calcium ionophores require the atypical PKCζ isoform for the induction of histone citrullination and NETosis (Neeli and Radic, 2013). Interestingly, PMA not only fails to promote histone citrullination but also inhibits the histone citrullination induced by a calcium ionophore by means of PKCα/β activity (Neeli and Radic, 2013). Thus, divergent programs of NETosis can be initiated through the activity of distinct PKC isoforms. A large screening of small-molecule compounds revealed that a selective inhibitor of c-Raf impaired PMA-induced NETosis at an early stage of nuclear disruption and chromatin expansion. Furthermore, PMA-induced NETosis involves PKC activation of the MEK/ERK pathway, with the c-Raf/MEK/ERK pathway upstream of NADPH oxidase activation (Hakkim et al., 2011).

## Eosinophil Extracellular Traps

The release of DNA extracellular traps by eosinophils (named EETs) was first described by Yousefi et al. (2008), 4 years after the characterization of ET release from neutrophils. In the first study, the authors observed multiple extracellular DNA fibers associated with MBP and ECP in colon biopsy samples taken from patients with Crohn’s disease, schistosomiasis, or intestinal spirochetosis (Yousefi et al., 2008). *In vitro*, it was observed that human eosinophils primed with IFN-γ or IL-5 and activated with LPS, C5a, or eotaxin/CCL11 released EETs through a ROS-dependent mechanism. Interestingly, the origin of the DNA in the EETs was characterized as mitochondrial, and the phenomenon did not involve cell death (Yousefi et al., 2008). Yet in this same study, the authors showed that human eosinophils released EETs in response to opsonized *Escherichia coli*, and these EETs presented bactericidal activity. A few years later, a subsequent study showed that the stimulation of human eosinophils with thymic stromal lymphopoietin (TSLP), a cytokine secreted by epithelial cells and known to contribute to the promotion of Th2 responses, also induced the release of EETs of mitochondrial origin in a mechanism independent of cell death but dependent on the activation of NADPH oxidase and a β_2_-integrin (Morshed et al., 2012). Non-cytolytic mitochondrial EET formation occurred independently of autophagy in eosinophils primed with GM-CSF and stimulated with C5a or LPS or with low concentrations of PMA without cell priming (Germic et al., 2017).

Afterward, the process of EET release involving cell death was described, introducing the concept of EETosis (similar to the mechanism observed for neutrophils), where eosinophils undergo a cytolytic process with nuclear disruption, DNA mixing with intact granules and release of chromatin and associated granules into the extracellular medium in an NADPH oxidase-dependent mechanism (Ueki et al., 2013). In this study, the EETs released by human eosinophils were observed *in vitro* after stimulation with immobilized immunoglobulins (IgG and IgA), PAF, calcium ionophore or PMA. The DNA that constituted the EETs was characterized as having nuclear origin, and histones were found to be associated with these EET structures. Thus, EETs can be either released from live eosinophils, after mitochondrial DNA mobilization, associated with ECP and EPO (Yousefi et al., 2008), or as part of a slower process that results in the death of eosinophils by cytolysis and extrusion of histone-enriched nuclear DNA associated with clusters of intact granules (Ueki et al., 2013).

Eosinophil extracellular traps are formed in either an oxidative NADPH oxidase-dependent or oxidative-independent manner. NADPH oxidase-dependent mechanisms are required for mitochondrial EET formation in response to IFN-γ/IL-5 cell priming followed by stimulation with LPS, C5a or eotaxin/CCL11 (Yousefi et al., 2008). The release of nuclear-derived EETs in response to PAF, IgG/IgA immune complexes or PMA also relied on ROS generation by NADPH oxidase as did EET formation induced by TSLP (Yousefi et al., 2008; Morshed et al., 2012; Ueki et al., 2013). However, lysophosphatidylserine (LysoPS) induces EET formation by a ROS-independent mechanism (Kim et al., 2020). As observed for neutrophils (Asaga et al., 2001; Nakashima et al., 2002), human eosinophils also express the enzyme PAD4 (Asaga et al., 2001), and PAD4-mediated histone citrullination is necessary for LysoPS-induced EET formation (Kim et al., 2020). However, the role of PAD4-mediated histone citrullination in EET formation is unknown for several ETT inducers, such as PMA, immune complexes, PAF and monosodium urate crystals (Schorn et al., 2012; Ueki et al., 2013).

EETs and/or EETosis have been implicated in various eosinophil-associated allergic diseases, including rhinosinusitis with nasal polyps, eosinophilic esophagitis, allergic asthma, eosinophilic bronchopulmonary aspergillosis, eosinophilic otitis, and chronic obstructive pulmonary disease (Simon et al., 2015; Ueki et al., 2016; Uribe Echevarría et al., 2017; Muniz et al., 2018; Hwang et al., 2019; Persson et al., 2019). EETs have also been characterized as pro-inflammatory entities in non-allergic processes, such as atherosclerotic plaque formation and thrombosis (Marx et al., 2019), sepsis and colitis (Yousefi et al., 2008).

## NETs and Pathogenic Fungi

In the context of pathogenic fungi, the NET release phenomenon has been described for *Candida albicans* and other *Candida* spp. *A. fumigatus, Paracoccidioides brasiliensis, Scedosporium apiospermum*, and *Histoplasma capsulatum* (Urban et al., 2006; Bruns et al., 2010; McCormick et al., 2010; Svobodová et al., 2012; Mejía et al., 2015; Rocha et al., 2015; Campos-Garcia et al., 2019; Luna-Rodríguez et al., 2020; Negoro et al., 2020; Thompson-Souza et al., 2020). Fungi present great diversity in terms of interactions with host cells and tissue colonization, forms of development and immune evasion strategies. Neutrophils and other immune cells must deal with small fungal structures, such as conidia and yeast cells, which can be phagocytosed, as well as large multicellular forms, such as hyphae, that require non-phagocytic effector mechanisms. Most of the current knowledge on the induction of NETosis and EETosis is based on investigations using non-physiological inducers, such as PMA or calcium ionophores, which are useful for the elucidation of the basic mechanisms of NET formation, but do not account for the true complexity of pathogens. In this sense, fungal pathogens have provided valuable information about the mechanisms of ET formation and the roles of ETs in immunity and pathology.

## *Candida albicans-*Induced Net Formation

The dimorphic fungus *C. albicans* is a component of the human microbiota colonizing tissues such as skin, the genitourinary tract, gastrointestinal tract and oral mucosa. *C. albicans* is the major causative agent of mucosal fungal infections in healthy individuals and a relevant pathogen causing life-threatening disseminated infections in immunocompromised patients worldwide, particularly neutropenic patients (Poulain, 2015). *C. albicans* hyphae and yeast cells induce NET release in human neutrophils, and these extracellular traps ensnare and kill both morphotypes (Urban et al., 2006). While *C. albicans* hyphae are efficient inducers of NETosis, in the absence of the morphological transition to hyphae, yeast cells are unable to induce NET release (Branzk et al., 2014; Guiducci et al., 2018; Wu et al., 2019). The inability of *C. albicans* yeast cells to induce NETosis is a consequence of yeast phagocytosis, which reroutes NE from azurophilic granules to phagosomes, while hyphae that are not internalized trigger NET formation, with the release of NE to the cytosol and its subsequent nuclear translocation, leading to the NE-mediated degradation of histones (Branzk et al., 2014). NETs contain associated calprotectin, a cytosolic protein released during NETosis but not during neutrophil degranulation (Urban et al., 2009). NET-associated calprotectin chelates zinc, which inhibits *C. albicans* growth, and immunodepletion of calprotectin abolishes the fungicidal effect of NETs on *C. albicans*. Corroborating the role of calprotectin *in vitro*, calprotectin-deficient mice show increased susceptibility in experimental models of *C. albicans* infection (Urban et al., 2009).

Different mechanisms have been proposed for *C. albicans*-induced NET formation. NETosis induced by *C. albicans* in mouse neutrophils requires an oxidative burst by NAPDH oxidase, as demonstrated by the impaired NET release from *gp91*^–/–^ neutrophils in response to *C. albicans* hyphae (Ermert et al., 2009). *C. albicans*-induced NETosis by human neutrophils requires the generation of ROS; however, neutrophils obtained from CGD patients show similar NET formation. The requirement for a ROS-dependent mechanism for *C. albicans*-induced NETosis was attributed to ROS generation by opsonized *C. albicans*, as the preincubation of *C. albicans* hyphae with a ROS scavenger abolished NET release; thus, ROS generation by *C. albicans* cells can supply the oxidant environment necessary for the induction of NETosis (Kenny et al., 2017). NADPH oxidase-independent NETosis has been described for *C. albicans* in the absence of serum opsonization by a mechanism involving Dectin-2 recognition, Syk, PKCδ, calcium mobilization and PAD4-mediated histone citrullination (Wu et al., 2019). Adding more complexity to the role of NADPH oxidase-dependent ROS generation in *C. albicans*-induced NETosis, a faster process (30–50 min) for NET formation has been described for neutrophils adhered to fibronectin in response to β-glucans or *C. albicans* (Byrd et al., 2013). Crosstalk involving fibronectin-derived signals and β-glucan recognition by Mac-1/CR3 results in NET formation via a ROS-independent pathway in response to β-glucans and *C. albicans* (Byrd et al., 2013). Thus, neutrophils exhibit flexible mechanisms for NET formation. In the absence of serum opsonization, Dectin-2 must recognize α-mannans on the surface of *C. albicans* hyphae (Saijo et al., 2010); in contrast, in the presence of serum opsonins, *C. albicans* recognition by Mac-1 leads to NADPH oxidase-dependent NETosis (Wu et al., 2019). The opsonins involved in the NETosis in response to *C. albicans* are unknown. Possible candidates for *C. albicans* opsonization for NET formation include iC3b or fibrinogen, well-established ligands for Mac-1 (Diamond et al., 1993). Furthermore, in the context of extracellular matrix adhesion upon tissue migration, signaling by fibronectin recognition must be associated to the direct recognition of fungal β-glucans by Mac-1 to trigger NET release in the absence of an oxidative burst.

**FIGURE 1:**
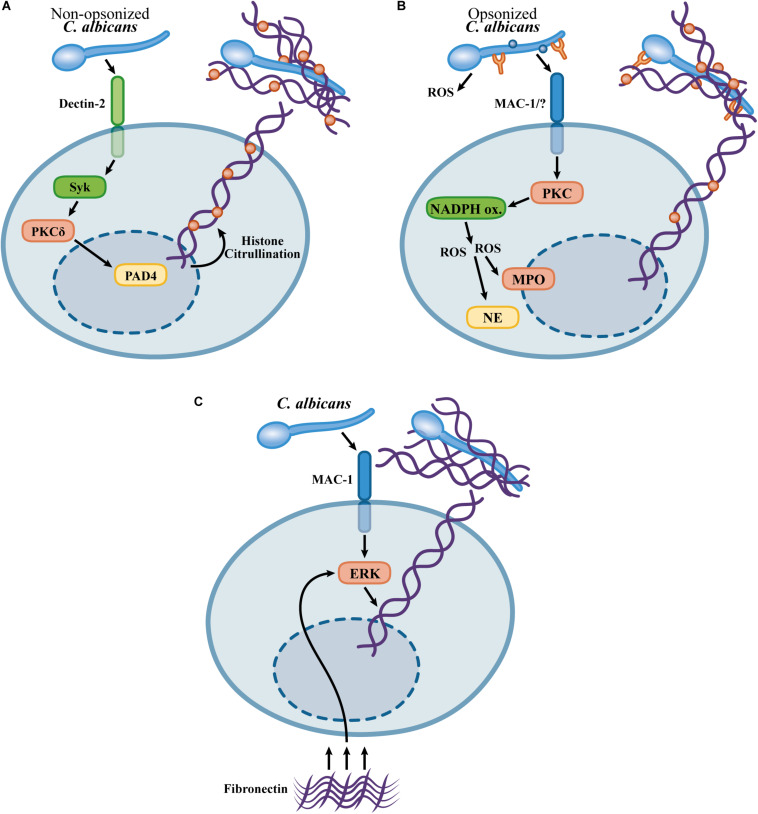
Mechanisms proposed for NET formation in response to *Candida albicans*. **(A)** Dectin-2 mediates NETosis in response to non-opsonized *C. albicans* via a pathway involving Syk, PKCδ, and the PAD4-mediated histone citrullination (Wu et al., 2019). **(B)** Opsonized *C. albicans* triggers NETosis by a ROS-dependent pathway involving NADPH oxidase activity (mouse neutrophils) (Ermert et al., 2009) or ROS generation by *C. albicans* (human neutrophils) (Kenny et al., 2017). PAD4-mediated citrullination is not required for NETosis in response to opsonized *C. albicans*, however, histone citrullination occurs during NETosis (Kenny et al., 2017; Guiducci et al., 2018). **(C)** Fast NETosis induced by the crosstalk upon the adhesion to fibronectin and recognition of *C. albicans* β-glucans by Mac-1 (Byrd et al., 2013).

The contribution of the distinct mechanisms for NETosis in the context of *C. albicans* infection remains a point of discussion. In an experimental model of *C. albicans* peritonitis, pharmacological inhibition of PAD4 abolished NET formation *in vivo*, leading to *C. albicans* dissemination to the kidneys (Wu et al., 2019). In contrast, in an intravenous model of *C. albicans* infection, PAD4-KO mice showed a slight increase in *C. albicans* load in the kidneys without increased kidney pathology or increased fungal burden at later timepoints, indicating that PAD4 is dispensable for immunity in a model of systemic candidiasis (Guiducci et al., 2018). Moreover, PAD4-deficient neutrophils show similar fungicidal activity and NETosis in response to *C. albicans* (Guiducci et al., 2018). The mechanistic differences in *C. albicans*-induced NETosis *in vivo* are not clear, but it is possible that, in the context of a local infection, in the absence of *C. albicans* opsonization, the Dectin-2/PAD4-dependent pathway is critical for NETosis (Wu et al., 2019), while a PAD4-independent mechanism leads to NET formation in systemic infection in which *C. albicans* must be exposed to serum opsonins (Guiducci et al., 2018). Figure 1 summarizes the mechanisms described for *C. albicans*-induced NET release.

## *Aspergillus fumigatus*-Induced NETosis

*Aspergillus* spp. are saprophytic mold fungi with a worldwide distribution. *Aspergillus* species produce small spores (2–3 μm), conidia, which are critical for the fungal dispersion. Under adequate conditions, conidia germinate, giving rise to the filamentous multicellular morphotype, the hyphae. *Aspergillus* spp. conidia are easily dispersed in the air which results in continuous human exposure to the inhalation of these conidia (Latgé and Chamilos, 2019). *A. fumigatus* is an environmental fungus and is also a common opportunistic agent causing invasive human respiratory diseases, such as allergic bronchopulmonary aspergillosis (ABPA) and a severe pulmonary infection, invasive aspergillosis (IA) (Kosmidis and Denning, 2015). The major predisposing factors for the development of invasive aspergillosis are immunosuppression associated with neutropenia or deficiencies in neutrophil effector functions (Brown et al., 2012; Gazendam et al., 2016a).

*Aspergillus fumigatus* resting conidia, swollen conidia, germ tubes and hyphae induce the formation of NETs (Bruns et al., 2010; McCormick et al., 2010). Web-like DNA structures are also formed in the lungs of *A. fumigatus*-infected mice and are absent in neutrophil-depleted mice, indicating that they are a consequence of NETosis *in vivo* and not a result of cell damage during infection (Bruns et al., 2010). In contrast to the NET formed against *C. albicans*, the NETs are not fungicidal for *A. fumigatus* or *A. nidulans* conidia; instead, these NETs exert fungistatic effects that have been attributed to zinc chelation by calprotectin (McCormick et al., 2010; Bianchi et al., 2011). Neutrophils cause a slight decrease in the metabolic rate of *A. fumigatus* hyphae, and DNAse I addition restores hyphal metabolic activity (Bruns et al., 2010). Thus, it seems that although they do not play roles in the killing of *A. fumigatus*, NETs must inhibit fungal growth and germination. Furthermore, NETs capture *A. fumigatus* conidia and hyphae, which prevents tissue dissemination. Interestingly, two-photon confocal analyses reveal neutrophils carrying swollen conidia and small hyphae in proximity to other neutrophils, which results in the entrapment of fungal structures, a process that must maximize neutrophil effector activity, such as NETosis and oxidative killing (Bruns et al., 2010).

NETosis in response to *A. fumigatus* requires superoxide generation by the NADPH oxidase complex. In an experimental model of *A. fumigatus* pulmonary infection, NADPH oxidase-deficient (*Ncf1* KO, p47-deficient) mice did not form NETs in lung tissue, and p47-deficient neutrophils were unable to release NETs in response to *A. fumigatus* hyphae (Röhm et al., 2014). *In vitro* results confirmed the role of NADPH oxidase in NETosis in response to *A. fumigatus* conidia (Bruns et al., 2010; Silva et al., 2020). The role of NADPH oxidase in NETosis in response to *A. fumigatus* hyphae seems to differ according to the experimental conditions. Opsonization of *A. fumigatus* hyphae with human serum induces NET formation in a NADPH oxidase-independent way (Gazendam et al., 2016b), while in other experimental settings without human serum, NETosis in response to hyphae requires NADPH oxidase activity (Bruns et al., 2010).

TLR2, TLR4, Dectin-1, and the β_2_-integrin Mac-1 are involved in the immune recognition of *A. fumigatus* (Mambula et al., 2002; Meier et al., 2003; Hohl et al., 2005; Steele et al., 2005; Gersuk et al., 2006; Gazendam et al., 2016b). While, TLR2, TLR4, and Dectin-1 are involved in cytokine production by macrophages in response to *A. fumigatus*, these receptors are dispensable for NETosis induced by *A. fumigatus* (Clark et al., 2018; Silva et al., 2020). Mac-1 recognizes fungal β-glucans through a lectin domain distinct from the so-called I-domain involved in the recognition of iC3b, fibrinogen and ICAM-1 (Thornton et al., 1996; Xia and Ross, 1999). NETosis in response to the *A. fumigatus* hyphal extracts, and curdlan, a particulate preparation of β-glucans, requires the recognition mediated by Mac-1 through its lectin domain, as evaluated by the blockade with a monoclonal antibody that targets this domain (Clark et al., 2018). This mechanism differs from the mechanisms of NET release and ROS generation induced by *A. fumigatus* live conidia, which are mediated by Mac-1 through the I-domain without the participation of the lectin domain (Silva et al., 2020). The I-domain is the region in the α_M_ chain critical for binding to fibrinogen, ICAM-1 and iC3b (Diamond et al., 1993). Recognition of β-glucans by the lectin domain in Mac-1 promotes an active conformational change in the Mac-1 complex that shows increased binding through the I-domain. Priming of Mac-1 by the lectin domain also induces the exposure of an activation epitope in the I-domain that is the binding site of the monoclonal antibody CBRM1/5 (O’Brien et al., 2012). Thus, it seems possible that distinct mechanisms function during fungal recognition mediated by Mac-1: (1) a priming by the β-glucan recognition through the lectin domain that must promote the I-domain recognition of *A. fumigatus* and (2) direct recognition of *A. fumigatus* molecules by the I-domain in living conidia.

NETosis and neutrophil ROS generation induced by *A. fumigatus* conidia require the activity of Src kinases, Syk, and class I PI3Kδ (Silva et al., 2020). Mac-1 signaling involves the immunoreceptor tyrosine-based activation motif (ITAM)-containing membrane proteins DAP-12 and the FcRγ chain (Mócsai et al., 2006). Src kinases promote the phosphorylation of the ITAM motifs in DAP12 and the FcRγ chain, which recruits Syk, resulting in Syk phosphorylation (Mócsai et al., 2006). This signaling module is required for ROS generation and adhesion resulting from the β_2_-integrin adhesion (Lowell et al., 1996; Mócsai et al., 1999, 2006). Interestingly, neither Src kinases, Syk, DAP12 nor the FcRγ chain are required for chemotaxis and neutrophil migration *in vitro* and *in vivo* (Mócsai et al., 2002, 2006; Kovács et al., 2014), indicating that Src kinases and Syk are involved in specific neutrophil responses, such as ROS generation, adhesion and NETosis. Interestingly, Syk is required for NETosis in response to immune complexes and *S. aureus*, indicating that Syk is a convergent signaling molecule for NETosis induction by several stimuli (Van Ziffle and Lowell, 2009; Behnen et al., 2014).

Class I PI3Ks are involved in neutrophil responses, including oxidative burst, adhesion and chemotaxis (Hirsch et al., 2000; Sasaki et al., 2000; Sadhu et al., 2003; Boyle et al., 2011). Class IB PI3Kγ is activated downstream of G coupled-protein receptors (GPCRs), while class IA PI3 kinases α, β, and δ are activated by tyrosine kinase pathways (Luo and Mondal, 2015). Selective inhibition of class IA PI3Kδ abolishes ROS generation and NETosis in response to *A. fumigatus* conidia. In contrast, class I PI3Kγ inhibition exerts only partial effects on ROS generation and does not affect *A. fumigatus*-induced NET release at concentrations in which the selective effects of class I PI3Kγ are observed (Silva et al., 2020). NADPH oxidase activation in response to *A. fumigatus* hyphae requires the associated activity of class I PI3Kδ and β; however, the role of class I PI3Kβ in *A. fumigatus*-induced NETosis has not been established; thus, it remains to be investigated whether class IA PI3Kδ cooperates with another class IA PI3K to provide signaling for NETosis induction by *A. fumigatus* (Boyle et al., 2011). How class I PI3Ks trigger NETosis and ROS generation in response to *A. fumigatus* remain unclear. Class I PI3Ks phosphorylate membrane phosphoinositides (PIs) at the 3rd position of the inositol moiety, generating PtdIns(3,4,5)P_3_ (Luo and Mondal, 2015). Akt/PKB serine-threonine kinases are activated upon the translocation to the cell membrane and their PH domain interacts with PtdIns(3,4,5)P_3_ produced by the activity of class I PI3Ks (Luo and Mondal, 2015). In neutrophils, Akt2 phosphorylates and activates the NADPH oxidase subunit p47 in response to C5a and fMLP, which is required for the assembly of NADPH oxidase and its subsequent activity (Chen et al., 2010). In contrast, opsonized zymosan induces ROS generation in the absence of Akt activity, indicating that Akt activation must not be a general mechanism for the NADPH oxidase activation (Chen et al., 2010). Thus, it remains to be established whether Akt isoforms are downstream effectors of class I PI3Ks for NETosis and ROS generation in response to *A. fumigatus* and other fungi.

**FIGURE 2:**
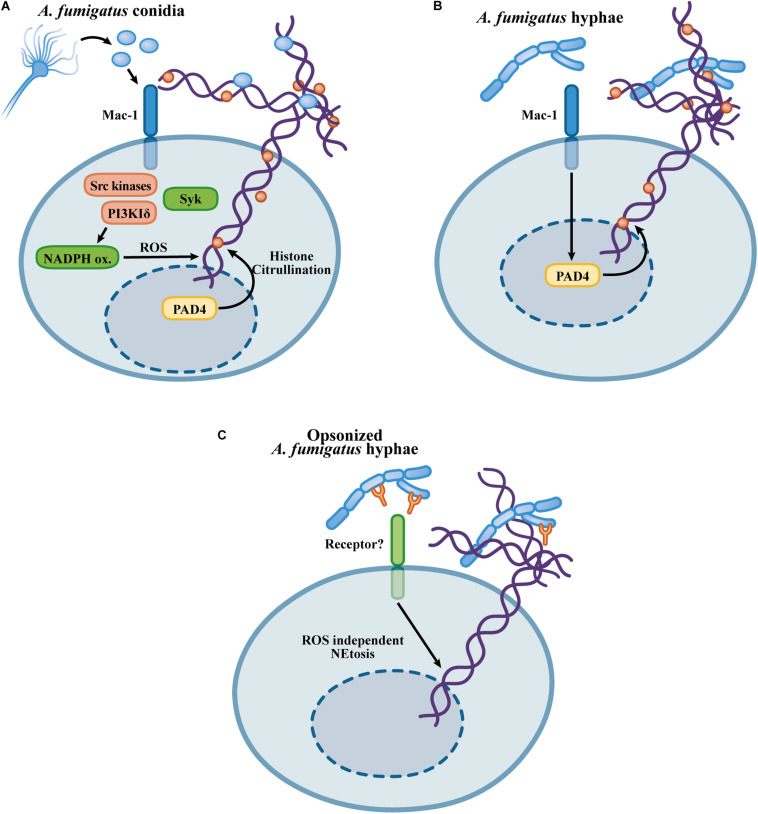
Mechanisms proposed for the NETosis in response to *Aspergillus fumigatus*. **(A)** Recognition of *A. fumigatus* triggers NETosis by a Mac-1/Src kinase/Syk and class I PI3Kδ mechanism, inducing a NET-releasing pathway that requires ROS generation by the NADPH oxidase but occurs independently of the PAD4-mediated histone citrullination (Silva et al., 2020). **(B)** *A. fumigatus* extracts, as well as β-glucans, induce NETosis through a Mac-1 and PAD4-dependent mechanism (Clark et al., 2018). **(C)** *A. fumigatus* opsonized hyphae promote NETosis through a NADPH oxidase-independent pathway (Gazendam et al., 2016b).

PAD4-mediated histone citrullination occurs during neutrophil activation with curdlan, a preparation of particulate β-glucans, *A. fumigatus* hyphae and conidia (Clark et al., 2018; Silva et al., 2020). NET formation in response to curdlan is partially dependent on histone citrullination mediated by PAD4, and in an experimental model of *A. fumigatus* corneal infection, histone citrullination was abolished in PAD4-KO mice (Clark et al., 2018). NET formation in response to *A. fumigatus* conidia, however, does not require PAD4 activity (Silva et al., 2020). The reason for the discrepant role of PAD4 in β-glucan- and *A. fumigatus*-induced NETosis is unknown. Since PAD4 histone citrullination makes only a partial contribution to β-glucan-induced NETosis, another pathway contributes to NET release (Clark et al., 2018). Furthermore, while curdlan is a particulate β-glucan preparation, *A. fumigatus* conidia express a variety of different molecules on their surface, which must trigger NETosis through a mechanism that overcomes a possible requirement for PAD4 activity. Figures 2A–C summarizes the mechanisms described for *A. fumigatus*-induced NET formation.

## NET Release in Response to the Dimorphic Endemic Fungi *H. capsulatum* var. *capsulatum* and *P. brasiliensis*

Histoplasmosis is an endemic disease whose etiologic agent is the fungus *H. capsulatum*, a thermally dimorphic fungus in the Americas and Africa that can affect both immunocompromised and immunocompetent individuals (Wheat et al., 2016; Oladele et al., 2018). *H. capsulatum* lives in the soil as a filamentous fungus. *H. capsulatum* mycelia produce sporulated structures, macroconidia and microconidia (8–14 and 2–5 μm diameter, respectively). Inhalation of *H. capsulatum* conidia and mycelial fragments results in pulmonary infections that can, in some cases, become disseminated, particularly in individuals presenting deficiencies in T-cell-mediated immune responses. Once in the host tissues at 37°C, *H. capsulatum* undergoes a morphological transition, giving rise to yeast cells that reside in an intracellular niche when phagocytosed by macrophages (Woods, 2016).

The interaction of neutrophils with *H. capsulatum* var *capsulatum* yeast cells results in phagocytosis and NET extrusion (Schnur and Newman, 1990; Newman et al., 1993, 2000; Thompson-Souza et al., 2020). Although neutrophils are capable of phagocytosing *H. capsulatum* cells, phagocytosis does not seem to underlie their antifungal activities (Brummer et al., 1991; Kurita et al., 1991; Newman et al., 1993). It was previously shown that *H. capsulatum* yeast cell phagocytosis by neutrophils triggers an oxidative burst process that is interestingly unable to contribute to fungal killing (Schnur and Newman, 1990; Kurita et al., 1991). The capacity of *H. capsulatum* to neutralize the fungicidal effect of the oxidative burst is attributed to its efficient system of antioxidative enzymes (Youseff et al., 2012; Holbrook et al., 2013). Another observation that corroborates this finding is that neutrophils obtained from patients with CGD show fungistatic activity similar to that of neutrophils that generate an oxidative burst (Newman et al., 1993). Neutrophils can bind opsonized *H. capsulatum* through Mac-1 (CD11b/CD18), CR1, and FcγRIII in a cooperative manner (Newman et al., 1993). As mentioned, ROS do not seem to be a relevant mechanism for *H. capsulatum* killing by neutrophils; however, components of primary granules such as cathepsin G, bactericidal/permeability-increasing protein (BPI) and defensins seem to play fungistatic roles (Newman et al., 1993, 2000). Interestingly, this fungistatic effect did not require uptake of yeast cells (Newman et al., 1993). Thus, phagocytosis does not explain the fact that neutrophils have antifungal capabilities against *H. capsulatum* yeast cells.

Thompson-Souza et al. described that *H. capsulatum* yeast cells induce NET release by human neutrophils, and the extracellular killing of *H. capsulatum* requires NET formation (Thompson-Souza et al., 2020). This process occurred through a signaling pathway mediated by NADPH oxidase-dependent ROS generation, β_2_-integrin-mediated recognition, Src kinases and Syk, and culminated in the loss of neutrophil viability. Neutrophil ROS production in response to *H. capsulatum* required Src kinase and Syk activity, demonstrating a role for NADPH oxidase-dependent oxidative burst downstream of the signaling cascade promoting NETosis (Thompson-Souza et al., 2020). In addition, NETs formed in response to *H. capsulatum* show bona fide NET markers, such as associated NE and citrullinated histones. However, extrusion of *H. capsulatum*-induced NETs occurs independently of PAD4-mediated histone citrullination (Thompson-Souza et al., 2020).

**FIGURE 3:**
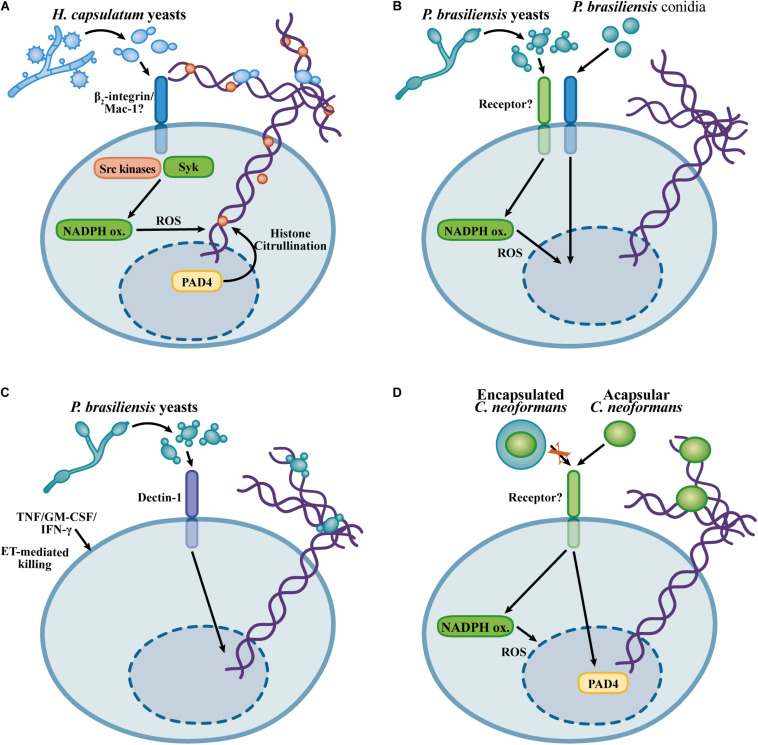
Overview of the mechanisms described for the NET induction in response to *Histoplasma capsulatum*, *Cryptococcus neoformans*, and *Paracoccidioides brasiliensis*. **(A)** *H. capsulatum* NETosis relies on β_2_-integrin recognition, Src and Syk kinase, via a NADPH oxidase-dependent process, but in a histone citrullination-independent manner (Thompson-Souza et al., 2020). **(B)** NET formation in response to *P. brasiliensis* yeast cells and conidia occurs through NADPH oxidase-dependent and NADPH oxidase -independent mechanisms, respectively (Mejía et al., 2015; Bachiega et al., 2016). **(C)** Dectin-1 mediated recognition of *P. brasiliensis* yeast cells promotes NET formation. Although *P. brasiliensis* yeast cells are resistant to the neutrophil killing, neutrophil-activating cytokines promote fungicidal activity against *P. brasiliensis* through NET-mediated fungal damage (Bachiega et al., 2016). **(D)** Mechanisms of NETosis in response to *C. neoformans*. Encapsulated *C. neoformans* evades NETosis, while acapsular yeast cells induce NET formation by NADPH oxidase- and PAD4-dependent mechanisms (Rocha et al., 2015).

The fact that NETs exhibit fungicidal activity against *H. capsulatum* may explain the apparent contradiction found by Newman et al. (1993) who showed that the uptake of yeast cells was not required for the antifungal activities of neutrophils. While previous works have evaluated total killing through neutrophil lysis and *H. capsulatum* CFU counts after relatively short periods of incubation, Thompson-Souza et al. (2020) investigated extracellular *H. capsulatum* killing by propidium iodide entry into yeast cells after 6 h. Thus, it seems that *H. capsulatum* yeast cells are able to survive the intracellular microbicidal activity of neutrophils, while the extracellular yeasts trapped by NETs are vulnerable to the toxic components present in the NETs. The presence of NETs, as well as the role of these structures, in infections caused by *H. capsulatum* have not been investigated; therefore, it remains to be established whether NETs contribute to the control of *H. capsulatum* in the context of infection or even whether NETs contribute to the immunopathogenesis during histoplasmosis. Figure 3A shows the mechanisms involved in *H. capsulatum*-induced NETosis.

*Paracoccidioides brasiliensis* is a pathogenic dimorphic fungus that causes paracoccidioidomycosis, a systemic disease prevalent in Latin America (Colombo et al., 2011). Infections caused by *P. brasiliensis* seem to be established upon the inhalation of conidia that subsequent differentiate in the host environment to establish intracellular yeast parasitism in macrophages (Cezar-Dos-Santos et al., 2020). Human neutrophils form NETs in response to *P. brasiliensis* conidia and yeast cells, and NETs are found in the lesions obtained from patients suffering from paracoccidioidomycosis (Della Coletta et al., 2015; Mejía et al., 2015). NETosis induced by *P. brasiliensis* yeast cells requires ROS generation by the NADPH oxidase complex, while conidia trigger NET release by an NADPH oxidase-independent mechanism. In any case, neutrophils are unable to kill *P. brasiliensis* yeast cells. Consistent with the lack of fungicidal activity of neutrophils, NET degradation by DNAse I, inhibition of NADPH oxidase or phagocytosis do not affect the viability of this fungus during the interaction with neutrophils (Mejía et al., 2015). NETosis in response to *P. brasiliensis* is partially dependent on Dectin-1, while TLR2 and TLR4 are dispensable (Bachiega et al., 2016). Although human neutrophils exhibit reduced fungicidal activity against *P. brasiliensis*, neutrophil primming with TNF, GM-CSF, or IFN-γ results in *P. brasiliensis* killing by a mechanism requiring NET formation (Bachiega et al., 2016). Thus, although *P. brasiliensis* evades neutrophil killing, their ability to escape from neutrophil fungicidal mechanisms can be overcome by neutrophil activation induced by pro-inflammatory cytokines, leading to NET-associated fungicidal activity. It would be interesting to evaluate whether pro-inflammatory cytokines lead to a different NET composition or whether cooperation of NETs with other microbicidal mechanisms can be induced by neutrophil-activating cytokines, thus enabling fungal killing. Figures 3B,C illustrates the findings concerning the NET formation in response to *P. brasiliensis*.

## *Cryptococcus gattii* and *Cryptococcus neoformans*

*Cryptococcus gattii* and *C. neoformans* are environmental fungi able to grow as encapsulated yeast cells. Infections caused by *C. neoformans* and *C. gattii* are initiated by the inhalation of environmental yeast cells or spores, which establishes a pulmonary infection and subsequent dissemination to the central nervous system leading to meningitis. While *C. gattii* has been identified as a primary pathogen causing infections in immunocompetent individuals, *C. neoformans* infections are mostly associated with deficiencies in T cell-mediated immunity, especially in HIV-infected patients (May et al., 2016). Human neutrophils exhibit fungicidal activity against *C. neoformans* by either oxidative or non-oxidative mechanisms (Mambula et al., 2000). Through the fractionation of human neutrophils, in cytosolic and granular preparations, the antifungal components calprotectin and defensins (human neutrophil proteins 1 and 3, HNP-1 and HNP-3), have been identified as anticryptococcal molecules present in the cytosol and primary granules, respectively (Mambula et al., 2000). Killing of *C. gattii* by neutrophils requires serum opsonization, phagocytosis and serine protease activity, but the NADPH oxidative burst is dispensable (Ueno et al., 2019).

*Cryptococcus gattii* yeast cells growing on plant-derived material produce extracellular fibrils. The formation of extracellular fibrils results in increased resistance of *C. gattii* yeast cells to killing by neutrophils. Paradoxically, the production of extracellular fibrils by *C. gattii* results in increased NET formation, indicating that although extracellular fibrils induce neutrophil activation, these structures confer resistance to neutrophil effector mechanisms (Springer et al., 2010). NETs show fungicidal activity against *C. neoformans* yeast cells due to the microbicidal effects of MPO, elastase, histones and collagenase (Urban et al., 2009; Rocha et al., 2015). While encapsulated *C. neoformans* yeast cells do not induce NET formation, a capsule-deficient strain leads to NETosis via an NADPH oxidase- and PAD4-dependent mechanism (Rocha et al., 2015). Glucuronoxylomannan (GXM) is the major component of the *C. neoformans* capsule (O’Meara and Alspaugh, 2012). Purified GXM inhibits NET formation and neutrophil ROS generation, and incubation of a non-encapsulated *C. neoformans* strain with GXM results in inhibition of NETosis, thus indicating that GXM is the component in the *C. neoformans* capsule critical for the inhibition of NETosis. Interestingly, glucuronoxylomannogalactan (GXMGal), a minor component of the *C. neoformans* capsule, is an inducer of NET formation through a ROS-independent mechanism (Rocha et al., 2015). Thus, the secretion of extracellular polysaccharides by *Cryptococcus* spp. represents a major evasion mechanism for the fungicidal activity of NETs. Figure 3D illustrates the knowledge about NET formation in response to *C. neoformans*.

## EETs and Fungi

Different studies have characterized eosinophils as capable of recognizing fungi, as well as fungal molecular patterns, which promote eosinophil activation and antifungal responses (Inoue et al., 2005; Yoon et al., 2008; Garro et al., 2011; Lilly et al., 2014). Human eosinophils respond to *A. alternata* hyphae by releasing their granular eosinophilic content, which reduces fungal viability (Yoon et al., 2008). Eosinophil degranulation was induced by β-glucans, but not chitin, through recognition mediated by the β_2_-integrin Mac-1 (Yoon et al., 2008). In addition to the recognition of β-glucans, eosinophils also recognize proteases secreted by *A. alternata* in a mechanism that involves protease-activated receptor-2 (PAR-2) (Matsuwaki et al., 2009, 2011). Eosinophils are required for the clearance of *A. fumigatus* in an experimental model of pulmonary infection (Lilly et al., 2014). In addition, *in vitro* murine bone marrow-differentiated eosinophils present fungicidal properties against *A. fumigatus* conidia in a mechanism that does not depend on contact (Lilly et al., 2014). Thus, despite different studies exploring the mechanisms of fungal recognition by eosinophils, there is still a shortage of evidence describing how eosinophils contribute to fungal infections by releasing EETs.

Bronchial secretions of patients with ABPA show EETs in association with large numbers of eosinophils with clear nuclear characteristics of EETosis. EETs in the mucus obtained from ABPA individuals show citrullinated histone 3 (Muniz et al., 2018). Human eosinophils form EETs in response to *A. fumigatus* conidia through a cytolytic process. EETs show labeling for MBP, a granular protein; however, in contrast to NETs that exhibit an association with free granule proteins, such as NE and MPO, EETs exhibit large punctuated immunostaining for cationic proteins, suggesting that these proteins are not freely attached to these structures (Muniz et al., 2018). The presence of clusters of intact free eosinophil granules associated with EETs has been described for other stimuli, such as a calcium ionophore and LysoPS (Ueki et al., 2013; Kim et al., 2020). Recent evidence indicates that intact granules are associated with *A. fumigatus*-induced EETs (Muniz et al., 2018), which must reflect a general mechanism by which many stimuli can lead to the cytolytic process in EET formation. In contrast, the presence of clusters of eosinophil granules attached to EETs was not observed in studies reporting mitochondrial-derived EET release (Yousefi et al., 2008; Morshed et al., 2012). In addition, there is no evidence showing that faster non-cytolytic mitochondrial EETs are released in response to fungal exposure. However, this is an interesting possibility that cannot be discarded.

In neutrophils, NE and MPO are directed to the nucleus and have roles in the chromatin decondensation that precedes nuclear membrane rupture, mixture of nuclear content and NET extrusion, as previously mentioned (Fuchs et al., 2007; Papayannopoulos et al., 2010). However, for eosinophils, it is uncertain whether granular proteins have roles in the chromatin decompaction and nuclear rupture that precede EETosis, or which molecules are involved in this process. Thus, the mechanisms that underlie EET extrusion considering the roles of granular proteins and possible mediators of EETosis remain to be elucidated.

Reactive oxygen species generation has traditionally been suggested as an important mechanism of host defense against pathogens and a downstream signal for the induction of NETosis (Fuchs et al., 2007; Winterbourn et al., 2016). However, pharmacological inhibition of NADPH oxidase or mitochondrial ROS generation does not interfere with *A. fumigatus*-induced EET formation, indicating that EETosis occurs independently of the major systems critical for ROS production in leukocytes (Muniz et al., 2018). Furthermore, eosinophils do not exhibit ROS production in response to *A. fumigatus*, while PMA is able to induce an oxidative burst in eosinophils in an NADPH oxidase-dependent manner. This difference is interesting because the release of EETs differs from NETs formed by neutrophils responding to *A. fumigatus*, and also from other neutrophil responses in which ROS involvement is clearly important (Bruns et al., 2010; Röhm et al., 2014; Winterbourn et al., 2016; Muniz et al., 2018; Silva et al., 2020).

Considerable discussion is reported regarding the receptors and proteins that may be involved in the recognition of the pathogens and signaling pathways that trigger the NET formation. In eosinophils, Mac-1 mediates the process of *A. fumigatus*-induced EET release (Muniz et al., 2018). Interestingly, Mac-1 emerges as a pattern recognition receptor (PRR) involved in neutrophil and eosinophil responses to fungal pathogens; however, some aspects remain uncharacterized, for example, (i) does Mac-1 directly recognize *A. fumigatus* molecules, and if it does, what is the identity of these molecules? (ii) Is there cooperation between Mac-1 and other PRR(s) during *A. fumigatus* recognition by neutrophils and eosinophils? Interestingly, peripheral human eosinophils do not express Dectin-1 (Yoon et al., 2008; Muniz et al., 2018), and neutralization of Dectin-1 does not impact the EET release induced by *A. fumigatus*, findings consistent with the inability of Dectin-1 to induce NET formation (Branzk et al., 2014; Muniz et al., 2018). However, it remains to be established whether there are roles for other well-established PRRs involved in fungal recognition, such as TLRs or other C-type lectin receptors (CLRs), in EETosis and NETosis.

**FIGURE 4:**
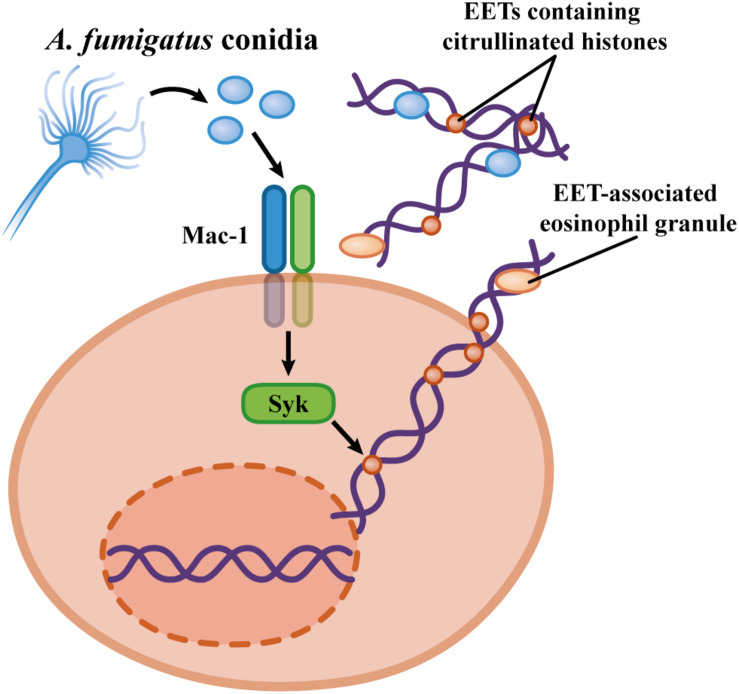
Mechanisms proposed by EET formation in response to *Aspergillus fumigatus*. Human eosinophils release ETs by a cytolytic process via the Mac-1 and Syk activation in a ROS-independent mechanism (Muniz et al., 2018).

Similarly, as observed for neutrophils, human eosinophils stimulated with *A. fumigatus* also release EETs in a Syk-dependent manner (Muniz et al., 2018; Silva et al., 2020). Investigations regarding the participation of other signaling components in the *A. fumigatus* stimulation of eosinophils are still needed. The roles of PAD4 and histone citrullination in EET release in response to *A. fumigatus* are also unknown. Furthermore, more investigations regarding the capacity of eosinophils to release EETs in response to other fungi, particularly those involved in ABPMs (such as *A. alternata* and *C. albicans*), are necessary. Figure 4 summarizes the findings on the mechanisms of *A. fumigatus*-induced EET formation.

## Mechanisms of Evasion of the Fungicidal Activity of ETs

Neutrophil extracellular trap formation is a relevant microbicidal mechanism of neutrophils, and many pathogenic fungi present mechanisms that prevent the induction of NETosis or attenuate the toxicity of NETs. Growth in a biofilm inhibits the killing of *C. albicans* hyphae by neutrophils (Johnson et al., 2016). *C. albicans* biofilms impair *C. albicans*-induced NETosis and ROS generation by neutrophils, as well as NET formation and ROS generation in response to PMA. The inhibitory activity of the *C. albicans* biofilm on neutrophil effector responses, such as NETosis and ROS generation, requires intact mannosylation of *C. albicans*. The mutant *C. albicans* strain *PMR1^Δ/Δ^* lacks a Ca^+2^-ATPase necessary for the activity of mannosyltransferases in the Golgi complex, which results in the formation of truncated mannan chains. Neutrophils show increased NET formation in response to *PMR1^Δ/Δ^ C. albicans* compared with wild-type *C. albicans*, and this phenotype is reversed by the restoration of mannosylation upon *PMR1* expression. Thus, the *C. albicans* biofilm seems to down modulate neutrophil signaling pathways possibly downstream of PKC activation, thus reducing the impact of NET toxicity (Johnson et al., 2016).

Preventing NET formation has also been observed in an encapsulated *C. neoformans* strain that does not induce NET release, in contrast with a non-encapsulated *C. neoformans* strain that promotes extensive NETosis and ROS generation. Interestingly, purified GXM inhibits PMA-induced NET formation and renders an acapsular *C. neoformans* strain unable to induce NET formation, thus indicating that, similar to the *C. albicans* biofilm, GXM acts as a secreted fungal molecule that inhibits neutrophil responses (Rocha et al., 2015). The expression of an external layer of hydrophobic proteins on *A. fumigatus* conidia conceals the ligands for PRRs, which decreases the release of NETs (Bruns et al., 2010) and the activation of PRRs, such as TLRs and Dectin-1 (Aimanianda et al., 2009). Thus, fungal pathogens evade the toxic effects of neutrophils by the secretion or surface expression of components able to inhibit NETosis induction.

The exopolysaccharide galactosaminogalactan (GAG) is found in the *A. fumigatus* cell wall, and this polysaccharide is composed of galactose and N-acetylgalactosamine (GalNAc) (Fontaine et al., 2011). The expression of GAG by *A. fumigatus* mediates its adherence and is required for full virulence and biofilm formation (Fontaine et al., 2011; Gravelat et al., 2013). *Aspergillus* species with lower pathogenicity, such as *A. nidulans*, present decreased expression of GalNAc in their GAGs, while *A. fumigatus* naturally produces GAG with higher amounts of GalNAc (Lee et al., 2015). The generation of *A. nidulans* strains overexpressing the enzymes involved in the synthesis of GalNAc results in increased resistance to neutrophil killing. This reduced susceptibility caused by the increased content of GalNAc in the *A. nidulans*-derived GAGs is a consequence of the resistance to the toxic effects of NETs, as indicated by DNAse treatment resulting in similar susceptibility of wild-type *A. nidulans* (Lee et al., 2015). The overexpression of GalNAc in *A. nidulans* strains resulted in increased virulence in murine experimental models of aspergillosis, which was similar to the virulence of *A. fumigatus* under the same experimental conditions (Lee et al., 2015). Thus, the expression of GAGs with a high content of GalNAc seems to be a determinant for the virulence of *A. fumigatus*. A recent paper demonstrated the activation of the NLRP3 inflammasome by *A. fumigatus* GAGs. The activation of the NLRP3 inflammasome by GAG was required for the control of infection in experimental models of aspergillosis, with an *A. fumigatus* strain deficient in GAG production showing increased virulence. Interestingly, the activation of the NLRP3 inflammasome by GAGs depended on the degree of N-acetylation, with acetylated GAGs unable to promote inflammasome activation (Briard et al., 2020). Thus, recognition of *A. fumigatus* GAGs by the NLRP3 inflammasome may reflect a mechanism for detecting a virulence factor produced by *A. fumigatus*; in contrast, increases in the degree of acetylation of GAGs may contribute to the evasion from the toxic effects of NETs and inflammasome activation. Therefore, it would be interesting to evaluate whether differences in GAG acetylation in clinical isolates of *A. fumigatus* are correlated with virulence.

The production of nucleases is a mechanism used by bacterial pathogens (Buchanan et al., 2006; Berends et al., 2010) and *Leishmania infantum* (Guimarães-Costa et al., 2014) to degrade NETs, thus avoiding neutrophil-mediated killing. *C. albicans* strains produce DNAse activity during interactions with neutrophils, and there is a correlation between the amount of DNAse produced and the susceptibility of different *C. albicans* strains to killing by neutrophils (Zhang et al., 2017). This work, however, did not identify the putative *C. albicans* DNAse(s) or offer formal proof using *C. albicans* mutants with deficient DNAse activity. Therefore, it remains to be established whether the production of DNase enzymes is a mechanism employed by pathogenic fungi to evade NET-induced toxicity.

## Roles of DNA Extracellular Traps in Fungal Infections

Neutrophil extracellular traps are mediators of the antimicrobial arsenal of neutrophils and have been implicated in the elimination and control of many pathogens (Brinkmann and Zychlinsky, 2012). In addition, many investigations have demonstrated a role for NETs in the pathogenesis in many experimental models and human diseases. NET-induced pathology has been observed in autoimmune diseases (Hakkim et al., 2010), atherosclerosis (Warnatsch et al., 2015), and thrombosis (Fuchs et al., 2010). Thus, the roles of NETs in fungal infections must involve both antifungal immunity and immunopathology. While NETs have been demonstrated to contribute to the antifungal activity of neutrophils *in vitro*, the roles of NETs in fungal infections are still poorly characterized. NETs have been described in experimental models of aspergillosis, scedosporiosis and candidiasis (Urban et al., 2009; Bruns et al., 2010; Wu et al., 2019; Luna-Rodríguez et al., 2020).

Using different experimental models of *C. albicans* infection, Urban et al. (2009) revealed that NETs are formed in infected tissues and restrict *C. albicans* spread. Calprotectin-deficient mice show increased susceptibility in experimental models of cutaneous, pulmonary, and systemic *C. albicans* infection, which is manifested by increased fungal loads (pulmonary infection), lethality (pulmonary and systemic infections), and formation of skin abscesses (Urban et al., 2009). The increased susceptibility of calprotectin-deficient mice does not result from inefficient neutrophil tissue recruitment or NET formation, indicating that the effect of calprotectin is a consequence of its intrinsic antifungal activity (Urban et al., 2009). Since calprotectin is released in association with NETs and is also required for NET antifungal activity, the role of calprotectin in the immune response to *C. albicans* must reflect the fungicidal activity of NET-associated calprotectin. However, Urban et al. (2009) did not evaluate the formal roles of NETs *in vivo*, for example, by NET removal by DNAse *in vivo* treatment; therefore, it remains possible that calprotectin acts in a NET-independent way, as a fungicidal molecule. In an experimental model of intraperitoneal infection by *C. albicans*, NETs were formed, and the degradation of these NETs by the intraperitoneal administration of micrococcal nuclease (MNase) resulted in *C. albicans* dissemination to the kidneys in association with reduced *C. albicans* loads in the peritoneal cavity, which indicates that the peritoneal entrapment of *C. albicans* by NETs prevented the systemic spread of infection (Wu et al., 2019).

Although NETs are formed in the lung parenchyma in a murine model of experimental aspergillosis (Bruns et al., 2010; Röhm et al., 2014), their roles in immunopathogenesis remain unclear. In a murine model of pulmonary aspergillosis, PAD4-KO mice showed decreased fungal loads and alveolar edema; however, neutrophil recruitment and pulmonary pathology were not affected by PAD4 deficiency (Alflen et al., 2020). The results obtained with PAD4-KO mice have been interpreted as evidence of roles for NETs in experimental pulmonary aspergillosis. These conclusions, however, are based on the premise that PAD4 is required for *A. fumigatus*-induced NETosis, which has not been observed (Silva et al., 2020). Moreover, Alflen et al. did not formally evaluate the roles of NETs in the aspergillosis by determining whether (1) NETs were evident in the lung tissues of wild-type or PAD4-KO mice, (2) NETs were formed by wild-type or PAD4-KO neutrophils or whether (3) DNAse treatment had an effect in the outcome of infection (Alflen et al., 2020). NADPH oxidase deficiency resulted in the absence of *A. fumigatus*-induced NET release by murine neutrophils, and *Ncf1*^–/–^ mice showed the absence of NETs in lung tissues in an experimental model of pulmonary aspergillosis (Röhm et al., 2014). NADPH oxidase-deficient mice are extremely susceptible to pulmonary aspergillosis; however, the role of NADPH oxidase in aspergillosis is complex and must involve oxidative killing of *A. fumigatus*, as well as the control of lung inflammation, and probably does not reflect merely the absence of NETosis (Morgenstern et al., 1997; Winterbourn et al., 2016). A case study of gene therapy for CGD revealed that restoring NADPH oxidase activity in hematopoietic cells resulted in the recovery of disseminated infection by *A. nidulans*, which was associated with restored NETosis and the NET-mediated inhibition of *A. nidulans* germination (Bianchi et al., 2009). However, considering the complexity of this clinical study and the evident technical limitations, it is not possible to conclude a specific role for NETosis in CGD gene therapy, especially considering all the roles of NADPH oxidase in immunity. Interestingly, therapy with DNAse is an approach for the treatment of cystic fibrosis. *A. fumigatus* pulmonary infections are common conditions in individuals suffering from cystic fibrosis. A clinical study investigating risk factors for the development of pulmonary aspergillosis pointed to therapy with recombinant DNAse as a factor associated with the incidence of pulmonary aspergillosis in pediatric patients with cystic fibrosis (Jubin et al., 2010). Therefore, it seems possible that the continuous formation of NETs prevents *A. fumigatus* colonization of the airways in cystic fibrosis and other pulmonary conditions where mucus accumulation is present. Alternatively, NETs must promote tissue pathology, contributing to the severity of pulmonary aspergillosis. Thus, more detailed investigations of the role of NETs in experimental models of aspergillosis are necessary.

The roles of EETs in fungal infections are even more elusive. The bronchiolar mucus of patients with ABPA presents extracellular EETs and abundant infiltrates of eosinophils with clear nuclear characteristics of EETosis. Human eosinophils, however, do not show fungicidal/fungistatic effects on *A. fumigatus* conidia under experimental conditions in which EETosis occurs (Muniz et al., 2018). Thus, it is possible that EETs ensnare viable *A. fumigatus* conidia in the bronchial tree, which must cause chronic colonization of *A. fumigatus* and perpetuation of allergic inflammation. In a mouse model of ABPM induced by intranasal challenge with *A. fumigatus* conidia, eosinophils were dispensable for pathological alterations, such as mucus production and pulmonary edema. Although the absence of eosinophils did not interfere with IL-5 and IL-13 production, eosinophils were required for IL-4, IL-17, and IL-23 production (Dietschmann et al., 2020). Thus, eosinophils play immunomodulatory roles in the experimental ABPM, although some aspects of the pulmonary pathology occur independently of eosinophil activity. However, this work did not evaluate parameters such as fungal load, respiratory mechanics, airway remodeling, collagen deposition and formation of EETs. Thus, more investigations are necessary to establish the roles of EETs and eosinophils in ABPMs.

Signaling pathways involved in the formation of NETs and EETs are also implicated in other neutrophil and eosinophil responses, such as the modulation of apoptosis, microbicidal killing and production of inflammatory mediators. For example, NADPH oxidase is critical for microbial oxidative killing, as well as the modulation of the inflammatory response and neutrophil apoptosis (Winterbourn et al., 2016; Warnatsch et al., 2017). Thus, the use of NADPH oxidase-deficient mice would fail to reveal specific roles of NETs in experimental models of fungal infection. The same situation must occur in the context of PAD4, NE, and MPO deficiency. Therefore, complementary approaches must be used to establish the role of NETs in fungal infections, such as the use of DNAse *in vivo* treatment, use of experimentally induced neutrophil/eosinophil selective deficiency of signaling molecules or receptors, neutrophil/eosinophil transfer experiments, and the detection of NETs/EETs *in vivo*.

## Conclusion

Neutrophils are critical effectors of antifungal immunity, but they also contribute to the immunopathology associated with infections. Although the roles of eosinophils in fungal diseases are still poorly understood, growing evidence indicates that these leukocytes show antifungal activity and immunomodulatory mechanisms in fungal-associated pathologies. Neutrophils and eosinophils release ETs, structures that exhibit microbicidal actions but also promote tissue injury. Although considerable information has been obtained through *in vitro* experiments, NETosis and EETosis are still poorly characterized in experimental models of mycoses. Thus, the comprehension of the mechanisms involved in NETosis/EETosis in response to fungal pathogens must contribute for the design of new therapeutic strategies, including new pharmacological targets.

## Author Contributions

All authors listed have made a substantial, direct and intellectual contribution to the work, and approved it for publication.

## Conflict of Interest

The authors declare that the research was conducted in the absence of any commercial or financial relationships that could be construed as a potential conflict of interest.
